# Recent Developments on gMicroMC: Transport Simulations of Proton and Heavy Ions and Concurrent Transport of Radicals and DNA

**DOI:** 10.3390/ijms22126615

**Published:** 2021-06-21

**Authors:** Youfang Lai, Xun Jia, Yujie Chi

**Affiliations:** 1 Department of Physics, University of Texas at Arlington, Arlington, TX 76019, USA; youfang.lai@mavs.uta.edu; 2 Innovative Technology of Radiotherapy Computation and Hardware (iTORCH) Laboratory, Department of Radiation Oncology, University of Texas Southwestern Medical Center, Dallas, TX 75287, USA

**Keywords:** Monte Carlo simulation, GPU programming, DNA damage, proton transport

## Abstract

Mechanistic Monte Carlo (MC) simulation of radiation interaction with water and DNA is important for the understanding of biological responses induced by ionizing radiation. In our previous work, we employed the Graphical Processing Unit (GPU)-based parallel computing technique to develop a novel, highly efficient, and open-source MC simulation tool, gMicroMC, for simulating electron-induced DNA damages. In this work, we reported two new developments in gMicroMC: the transport simulation of protons and heavy ions and the concurrent transport of radicals in the presence of DNA. We modeled these transports based on electromagnetic interactions between charged particles and water molecules and the chemical reactions between radicals and DNA molecules. Various physical properties, such as Linear Energy Transfer (LET) and particle range, from our simulation agreed with data published by NIST or simulation results from other CPU-based MC packages. The simulation results of DNA damage under the concurrent transport of radicals and DNA agreed with those from nBio-Topas simulation in a comprehensive testing case. GPU parallel computing enabled high computational efficiency. It took 41 s to simultaneously transport 100 protons with an initial kinetic energy of 10 MeV in water and 470 s to transport 10${}^{5}$ radicals up to 1 µs in the presence of DNA.

## 1. Introduction

Understanding biological responses to ionizing radiation is of crucial importance for cancer treatment using radiotherapy. Mechanistic Monte Carlo (MC) simulation of radiation’s effect on DNA in a water medium is a promising tool for relevant studies after decades of development [1]. The central idea of such an approach is to obtain the initial DNA damage spectrum via mechanistic modeling of the radio-biological interactions at the atomic or molecular levels. This includes the development of track-structure codes [2,3,4,5,6,7,8,9,10,11,12,13,14,15] and the subsequent computation of DNA damages by incorporating DNA models [5,7,16,17,18]. The track-structure simulation can be divided into the simulations of the physical stage and the chemical stage. The physical-stage simulation deals with the ionization, excitation, and elastic scattering processes between the ionizing radiation particles and the water media and records the 3D coordinates of energy deposition events. The chemical-stage simulation computes how the chemical radicals, produced after the physical-stage simulation, diffuse and react mutually with the recording of the residual radicals’ positions. The positions of these energy deposition events and radicals are then utilized to compute the initial DNA damage sites, followed by an analysis to characterize DNA strand break patterns.

Although many developments have been performed to generate state-of-the-art mechanistic MC simulation tools, it is still necessary to further improve the simulation methods to accommodate different scenarios [8]. For instance, to make the code versatile for studies on the Oxygen Enhancement Ratio (OER) [19] and the Fenton reaction effect [20], it is desired to include more types of molecules other than free radicals generated by the initial radiation into the chemical-stage simulation. However, due to the computational complexity of the “many-body” problem and the long temporal duration of the chemical stage, a step-by-step simulation of these relevant processes on conventional CPU computational platforms can be extremely time consuming [21]. Under the constraint of computational resources, studies typically suffer from a restricted simulation region or a shortened temporal duration [22,23,24], limiting their broad applications.

To overcome these obstacles, Graphical Processing Unit (GPU)-based parallel computing can be a cost-effective option [25,26]. We developed an open-source, GPU-based microscopic MC simulation toolkit, gMicroMC [9], with the first version available on GitHub (https://github.com/utaresearch/gMicroMC (accessed on 17 June 2021)). We initially focused on boosting the chemical-stage simulation for radicals produced from water radiolysis, achieving a speedup of several hundred folds compared to CPU-based packages [27]. Later, we supported the physical track simulation for energetic electrons and implemented a DNA model of a lymphocyte cell nucleus at the base-pair resolution for the computation of electron-induced DNA damages [9]. Recently, we also included oxygen molecules in the chemical-stage simulation in a step-by-step manner, which enabled the study of the radiolytic depletion effect of dissolved oxygen molecules [28]. With these efforts, we were able to quantitatively study multiple critical problems that are computationally demanding. For example, we performed comprehensive simulations with gMicroMC to answer how uncertainties from the simulation parameters affect the accuracy of the final DNA damage computations [29]. We also studied the radiolytic depletion of oxygen under ultra-high dose rate radiation (FLASH) to investigate the fundamental mechanism behind FLASH radiotherapy with the developed oxygen module in gMicroMC [28].

In this work, we reported our recent progress on two new and important features that we recently introduced to gMicroMC, namely: (1) enabling the physical-stage simulations of protons and heavy ions; and (2) considering the presence of the DNA structure and its chemical reactions with radicals in the chemical-stage simulation. It was expected that the first feature would contribute greatly to the mechanistic study of particle irradiation, such as particle radiotherapy [30,31]. The presence of the DNA structure in the chemical-stage simulation will allow us to realistically describe the indirect DNA damage process. With the GPU acceleration, we were able to afford computationally challenging simulations that included detailed physics modeling and chemical reactions that spanned over a large temporal duration, enabling more realistic simulations of the relevant processes.

## 2. Materials and Methods

### 2.1. Cross-Sections for the Transport Simulation of Protons and Heavy Ions

When a proton or heavy ion moves through a medium, it interacts with the atomic electrons inside the medium [32]. Considering that there have been various models developed and implemented to describe this process, in this work, we focused on a novel implementation of existing models on GPU parallel computing platforms. Specifically, we only considered the interactions between particles and water molecules because this is representative of modeling the cell environment. We employed the Rudd model [33] to compute the ionization of a water molecule by a proton in the energy range from 10 eV to 1 TeV. We implemented the Plante model [34] and Dingfelder’s model [35] to simulate the excitation of a water molecule for protons with an energy above and below 500 keV, respectively. We also applied Booth’s empirical formula [36] to include the charge effect on the cross-section computation. Lastly, we used the charge scaling rule [37] to obtain the cross-sections for heavy ions based on those for a proton. To make the manuscript easy to follow, we briefly introduce these models in the following subsections.

#### 2.1.1. Ionization for Protons

An energetic proton could eject a secondary electron from different atomic subshells when it ionizes a water molecule. In the Rudd model [33,38], the partial Singly Differential Cross-Section (SDCS) can be described as:

(1) $$\frac{d\sigma_{i}^{ion}}{dw}=\frac{S_{i}}{B_{i}}\frac{\left(F_{1}\left(\nu \right)+wF_{2}\left(\nu \right)\right)}{{\left(1+w\right)}^{3}}\frac{1}{1+\exp\left[\frac{\alpha \left(w-w_{i}\right)}{\nu }\right]}$$

Here, *i* refers to the subshells of the water molecule, namely ${{}^{1}b}_{1}$, ${{}^{3}a}_{1}$, ${{}^{1}b}_{2}$, ${{}^{2}a}_{1}$, and ${{}^{1}a}_{1}$. $B_{i}$ is the binding energy for electrons on shell *i*. $w=\;E_{e}/B_{i}$, and $E_{e}$ is the energy of the secondary electron. $S_{i}=4\pi a_{0}^{2}*N_{i}*{\left(E_{R}/B_{i}\right)}^{2}$, where $a_{0}=5.3\times 10^{-11}\;m$ is the Bohr radius, $E_{R}=13.6\;$ eV is the Rydberg energy, and $N_{i}$ is the number of electrons on shell *i*. $\nu =\sqrt{T/B_{i}}$ denotes the scaled velocity of the projectile, with $T=\frac{m}{M}*E_{k}$. m and M are the masses of the electron and proton, while $E_{k}$ is the kinetic energy of the proton. $w_{i}=4\nu^{2}-2\nu -E_{R}/4B_{i}$ is the scaled cutoff energy, and α is a numerical parameter related to the relative size of the target molecule. The specific values for $B_{i},\;N_{i}$ and α are listed in Table 1. $F_{1}\left(\nu \right)$ and $F_{2}\left(\nu \right)$ are fitting functions, defined as:

(2) $$F_{1}\left(\nu \right)=L_{1}+H_{1}$$

(3) $$F_{2}\left(\nu \right)=\frac{L_{2}H_{2}}{L_{2}+H_{2}}$$

where:

(4) $$\begin{array}{c} L_{1}=\frac{C_{1}\nu^{D_{1}}}{1+E_{1}\nu^{D_{1}+4}}, \end{array}$$

(5) $$\begin{array}{c} H_{1}=\frac{A_{1}\ln\left(1+\nu^{2}\right)}{\nu^{2}+B_{1}/\nu^{2}}, \end{array}$$

(6) $$\begin{array}{c} L_{2}=C_{2}\nu^{D_{2}}, \end{array}$$

(7) $$\begin{array}{c} H_{2}=\frac{A_{2}}{\nu^{2}}+\frac{B_{2}}{\nu^{4}}. \end{array}$$

The values for the nine basic parameters $A_{1},\cdots ,E_{1}$ and $A_{2},\cdots ,D_{2}$ used in Equations (4)–(7) can be seen in Table 1. These values differed for inner shell orbitals and external orbitals, and an orbital was regarded as an inner one when its binding energy exceeded twice the binding energy of the least-tightly bound orbital [33].

From Equation (1), we can calculate the total cross-section for subshell *i* as:

(8) $$\sigma_{i}^{ion}=\int_{0}^{w_{m}}\frac{d\sigma_{i}^{ion}}{dw}dw,$$

where $w_{m}=\frac{E_{m}}{B_{i}}$ and $E_{m}$ is the scaled maximum transferable energy from the proton to the ejected electron. The relativistic expression of $E_{m}$ was given by Plante et al. [34] as:

(9) $$E_{m}=\frac{2mc^{2}\left(\gamma^{2}-1\right)}{1+2\gamma \left(\frac{m}{M}\right)+{\left(\frac{m}{M}\right)}^{2}},$$

with:

(10) $$\gamma =1+\frac{E_{k}}{Mc^{2}},$$

and *c* is the speed of light. The relativistic format for the scaled velocity ν is then written as:

(11) $$\nu^{2}=\frac{mc^{2}}{2B_{i}}\left[1-\frac{1}{\gamma^{2}}\right].$$

With the ionization model and parameters determined under both the relativistic and nonrelativistic formalism, we could integrate Equation (8) numerically to obtain the ionization cross-section table for different subshells of a water molecule in a broad proton energy range. In our implementation, we computed the table for proton energies ranging from 10 eV to 1 TeV with a 0.01 increment along the logarithmic scale. It is worth mentioning that the computation of the cross-section table only needed to be computed once in an offline manner and was stored in a data file that could be loaded to GPU memory for the query of the cross-sections of any incident energy. More details of this usage are given in Section 2.3.1.

#### 2.1.2. Excitation for Proton

Due to the lack of experimental data, different models could have differential configurations of the excitation pathways. In our implementation, we adopted the three-pathway model [39,40] containing ${\tilde{A}}^{1}B_{1}$ and ${\tilde{B}}^{1}A_{1}$ and plasma mode for protons with energy >500 keV and the model with the excitation channels of ${\tilde{A}}^{1}B_{1}$ and ${\tilde{B}}^{1}A_{1}$, Ryd A + B and Ryd C + D, and the diffusion band [35] for a proton energy <500 keV. Specifically, in the three-pathway model, the differential cross-section for the excitation channel *j* is expressed as:

(12) $$\frac{d\sigma_{j}^{exc}}{dW}=\rho \left(W\right)Wf_{j}\left(W\right)\ln\left(\frac{W}{Q_{min}}\right),$$

where:

(13) $$\begin{array}{c} \rho \left(W\right)=\frac{8\pi Z^{2}a_{0}^{2}}{mu^{2}}\frac{Ry^{2}}{W^{2}}, \end{array}$$

(14) $$\begin{array}{c} Q_{min}=2T{\left(\frac{M}{m}\right)}^{2}\left(1-\frac{1}{2}\frac{m}{M}\frac{W}{T}-\sqrt{1-\frac{m}{M}\frac{W}{T}}\right). \end{array}$$

Here, *j* denotes different excitation channels, namely ${\tilde{A}}^{1}B_{1}$ and ${\tilde{B}}^{1}A_{1}$, and plasma mode. *u*, *Z*, and *W* are the velocity, charge, and energy loss of the incident proton. Other parameters were the same as those used in Section 2.1.1. When $\frac{m}{M}\frac{W}{T}=\frac{W}{E_{k}}\ll 1$, Equation (12) can be simplified as:

(15) $$\frac{d\sigma_{j}^{exc}}{dW}=\rho \left(W\right)Wf_{j}\left(W\right)\ln\left(\frac{4T}{W}\right).$$

In the relativistic situation, $mu^{2}$ in Equation (13) can be expressed as:

(16) $$mu^{2}=mc^{2}\left[1-\gamma^{-2}\right].$$

$f_{j}\left(W\right)$, as a function of the excitation pathway *j*, has the form of:

(17) $$f_{j}\left(W\right)=\{\begin{array}{cc} f_{j}^{0}\sqrt{\alpha_{j}/\pi }e^{\left[-\alpha_{j}{\left(W-w_{j}\right)}^{2}\right]}, & \mathrm{if}\;j={\tilde{A}}^{1}B_{1},\;{\tilde{B}}^{1}A_{1}\\ f_{j}^{0}\alpha_{j}e^{x}/{\left(1+e^{x}\right)}^{2}, & \mathrm{otherwise} \end{array}$$

where $x=\alpha_{j}\left(W-w_{j}\right)$ and $f_{j}^{0}$, $\alpha_{j}$, and $w_{j}$ are parameters with their values summarized in Table 2. Under the assumption that the proton only loses a small portion of its kinetic energy to excite a water molecule, i.e., $\frac{W}{E_{k}}\ll 1$, Equation (15) can be used to calculate the total cross-section for excitation channel *j* as:

(18) $$\sigma_{j}^{exc}=\int_{W_{min}}^{W_{max}}\frac{d\sigma_{j}^{exc}}{dW}dW.$$

In principle, the upper and lower boundaries of the integration can be $E_{k}$ and zero. However, in practical usage, it is common to set $W_{max}=$50 eV and $W_{min}=$ 2 eV. The reason is that $\frac{d\sigma_{j}^{exc}}{dW}\left(W\right)$ drops to a negligible value when $W\notin [W_{min},\;W_{max}]$, and the boundary cutoffs also ensure a positive and convergent integrated total cross-section.

When a proton’s energy is smaller than 500 keV, the Born approximation is no longer a good approximation [35], and Equation (15) may have problem in evaluating the cross-sections. We then applied the semi-empirical model [35] to obtain the excitation cross-section for a low-energy proton as:

(19) $$\sigma_{j}^{exc}\left(E_{k}\right)=\frac{\sigma_{0}{\left(Za\right)}^{\Omega }{\left(E_{k}-E_{j}\right)}^{v}}{J^{\Omega +v}+E_{k}^{\Omega +v}}.$$

Here, *j* denotes the excitation channels ${\tilde{A}}^{1}B_{1}$ and ${\tilde{B}}^{1}A_{1}$, Ryd A+B and Ryd C+D, and the diffusion band. The corresponding energy loss $E_{j}$ of the proton is discrete instead of continuous. Further details of the model can be found in [35].

With the excitation cross-section given in Equation (18) and the relevant parameters determined, we could integrate it numerically to obtain the excitation cross-section table for different subshells of a water molecule at proton energies above 500 keV. Meanwhile, we could rely on Equation (19) to handle protons with energies below 500 keV. To make the cross-section data computed from the two models smoothly connected at the proton energy of 500 keV, we adjusted the obtained cross-section data as follows. We applied coefficients of 1.23 and 3.5 to the cross-section data for the ${\tilde{A}}^{1}B_{1}$ and ${\tilde{B}}^{1}A_{1}$ channels obtained from Equation (18) to make them smoothly connected to that obtained from Equation (19) at 500 keV for these two modes. We then multiplied 0.339 in plasma mode to make the total cross-section also smoothly connected. Similar to the strategy applied to obtain the ionization cross-section table, we also only needed to compute the excitation cross-section table once and stored it in a data file. Its usage on a GPU is also given in Section 2.3.1.

#### 2.1.3. Charge Effect

When charged particles travel through a water medium, except for ionizing or exciting the water molecule, they could also drag electrons from the medium to move with them, forming a reduced effective charge $Z_{\mathrm{eff}}<Z$. This effect is found reversely proportional to the kinetic energy of the incident particle. In our simulation, we adopted the empirical Booth model [36] to obtain the effective charge $Z_{\mathrm{eff}}$ as:

(20) $$Z_{\mathrm{eff}}=Z\left(1-\exp\left[-1.316y+0.112y^{2}-0.065y^{3}\right]\right),$$

where $y=100Z^{-2/3}\sqrt{1-{\left(1+E_{k}/\left(AMc^{2}\right)\right)}^{-2}}$ and *A* is the mass number of the particle. The correction is larger than 5% ($Z_{\mathrm{eff}}<0.95\cdot Z$) when y<2.172, which gives $E_{k}∼$ 18 MeV per nucleon for the Fe ion and 0.22 MeV for the proton.

#### 2.1.4. Cross-Section for Heavy Ions

Within the first-order plane-wave Born approximation, we could correlate the ionization and excitation cross-section for bare and sufficiently fast heavy ions to that of the proton by the scaling law. Specifically, for a heavy ion with velocity *u* and charge number *Z*, the doubly differential cross-section can be scaled from that of the proton with the same velocity *u* by a factor of $Z^{2}$ [37]:

(21) $$\frac{d^{2}\sigma_{ion}}{dWdQ}\left(u\right)=Z^{2}\times \frac{d^{2}\sigma_{proton}}{dWdQ}\left(u\right),$$

where *W* and *Q* refer to the energy transfer and the recoil energy, respectively. After integrating over *Q*, we could obtain the SDCS as a function of *W*. Considering that an ion of mass number *A* and kinetic energy $E_{k}$ has the same velocity with a proton of kinetic energy $E_{k,p}=E_{k}\frac{M}{M_{ion}}\approx \frac{E_{k}}{A}$, we could rewrite the scaling formula as a function of $E_{k}$ as:

(22) $$\frac{d\sigma_{ion}}{dW}\left(E_{k}\right)=Z^{2}\times \frac{d\sigma_{proton}}{dW}\left(E_{k}/A\right),$$

It holds for ions for both the nonrelativistic and relativistic formats. The electron attachment effect can be more significant for a heavy ion than for a proton of the same velocity since a heavy ion typically carries more charge than a proton. With the electron attachment effect considered, we replaced *Z* with $Z_{\mathrm{eff}}$ when scaling the cross-section from a proton to a heavy ion using Equation (22), with $Z_{\mathrm{eff}}$ calculated from Equation (20).

### 2.2. Concurrent Transport Method

Due to the computational challenge, existing MC tools compute the DNA damage formed by radical attachment typically via two successive steps. First, the radicals are diffused and mutually reacted in the chemical stage without DNA. Second, the coordinates of OH· radicals obtained at the end of the chemical stage are overlapped with the DNA geometry such that DNA damages caused by radicals can be computed [9,29]. We refer to this approach as the “overlay method”. This approach is effective for simple applications. However, it can be problematic for those scenarios sensitive to radical evolution. In reality, DNA could be present and react with radicals during the radical diffusion. This could affect the radical yields and the damage site distribution on the DNA chain, consequently impacting the final characterization of the DNA strand breaks. To model this effect, in this work, we included the simulation of the reactions between radicals and DNA at the time of transporting the radicals in the chemical stage and refer to this approach as the “concurrent transport method”.

In our previous development of gMicroMC without considering DNA in the chemical stage, we simulated the diffusion and reactions among radicals in a step-by-step fashion. The relevant parameters were the diffusion coefficient for each radical species and the reaction rates for possible radical–radical reaction types. To include DNA in this transport frame, we need to know the diffusion coefficient of DNA and the reaction rates between radicals and DNA. Considering the relatively large mass of DNA, we assumed that the whole DNA molecule was static during the chemical transport and took its diffusion coefficient as zero to simplify the simulation. As for the reaction rates between radicals and DNA, we considered two types of reactions based on the DNA model for a whole lymphocyte cell nucleus [9]. The DNA was described in a voxel-based format with each voxel of side length 55 nm. The voxel was either empty or filled with a DNA chain that connected two faces of the voxel. The DNA chain consisted of a group of spheres representing the basic structures of the DNA: the base pair, the sugar-phosphate group, and the histone protein. With it, we considered the first reaction type as the damaging effect of OH· and $e_{h}$ radicals on the DNA bases and sugar-phosphate groups. The reaction rates are listed in Table 3. Here, although there were four types of DNA bases, that is Adenine (A), Guanine (G), Cytosine (C), and Thymine (T), associated with four different reaction rates with the OH· or $e_{h}$ radical, we used the average reaction rate in our simulation since our DNA model had no differentiation among the base types. The second type was the scavenging reaction for all radical species by the histone protein. In this reaction, the radical was assumed to be fully absorbed once it was within the histone protein volume.

After introducing DNA into the chemical-stage simulation, two consequences required special attention. First, radicals were not supposed to be produced inside the DNA region; hence, at the beginning of the chemical stage, we needed to exclude those radicals produced inside the chromatin zone from the subsequent diffusion [42] without recording any damages on DNA. Second, as there were a huge number of DNA basic structures in our DNA model, for instance $6.2\times 10^{9}$ base pairs, checking for reactions between radicals and DNA after each diffusion step would generate numerous computations. To circumvent the problem, we defined a time interval $t_{i}$ to control the frequency of checking for reactions between radicals and DNA. During the simulation of the chemical stage, as the time evolved, we only checked for radical–DNA reactions every $t_{i}$. In the limit of a small $t_{i}$, the frequent inspection for reactions ensured simulation accuracy. In the other extreme of a large $t_{i}$, the DNA-related reactions would be less frequently checked, which eventually converged to the overlay picture. We study the impact of $t_{i}$ in later sections.

### 2.3. GPU Implementation

#### 2.3.1. Physical Transport for Protons and Heavy Ions

Before transporting protons and heavy ions on the GPU, we prepared lookup tables on the CPU host to store the tabulated cross-sections for a proton, as stated in Section 2.1. The lookup tables were then transferred to the GPU texture memory such that we could employ the GPU built-in fast interpolation technique to obtain cross-section data for particle transport. We supported two types of source particle generation: reading from a Phase Space File (PSF) or sampling from a given distribution. We sorted the source particles in descending order based on their charge number *Z*. If the particles were protons or heavy ions, we transported the sorted particles into groups using the GPU kernel we developed in this work dedicated to proton and heavy ion transport. If they were electrons, we transported them with our previously developed kernel for electron transport [9]. The purpose of particle sorting and grouping was to minimize the thread divergence on the GPU, and hence to improve simulation efficiency [25,26].

For the GPU kernel in charge of the transport of protons and heavy ions, each thread took care of one primary particle. For a particle with charge number *Z*, atomic mass number *A*, and incident energy $E_{k}$, the thread sampled its free travel distance *s* in water and its interaction with the water molecules in the iteration. Specifically, we first calculated the effective charge number $Z_{\mathrm{eff}}$ according to Equation (20) and the kinetic energy $E_{p}=\frac{E_{k}}{A}$ for a proton with the same velocity as that of the primary particle. Based on the logarithmic value of $E_{p}$, we interpolated the lookup tables to obtain the cross-section $\sigma_{i}\left(E_{p}\right)$ for the proton. Here, *i* represents all ionization and excitation channels listed in the tables. We then scaled and summed $\sigma_{i}$ to obtain the total cross-section for the primary particle as $\sigma_{t}=Z_{\mathrm{eff}}^{2}\sum \sigma_{i}$ based on Equation (22). With $\sigma_{t}$, we sampled the free travel distance *s* in water as $s=-\frac{M_{w}}{\rho \cdot \sigma_{t}\cdot N_{A}}\ln\zeta$, where ρ and $M_{w}$ are the density and atomic mass of water. $N_{A}$ is the Avogadro constant, and ζ is a random number uniformly distributed between zero and one. We forwarded the particle position by *s* along the momentum direction followed by a sampling of the interaction type based on the relative cross-section distribution $\frac{\sigma_{i}}{\sum \sigma_{j}}$.

If the sampled interaction $i_{0}$ was an ionization event, we then sampled the energy $E_{e}$ and the emission angle of the ejected secondary electron, along with updating the kinetic energy of the primary particle. Noticing that the partial SDCS in Equation (1) had the form of $\frac{d\sigma_{i}^{ion}}{dw}\propto f\left(w\right)\phi \left(w\right)$, with $f\left(w\right)=\left(F_{1}\left(\nu \right)+wF_{2}\left(\nu \right)\right)/{\left(1+w\right)}^{3}$, which was relatively easy to integrate, and $\phi \left(w\right)=1/\left(1+\exp\left[\alpha \left(w-w_{i}\right)/\nu \right]\right)$, we took f(w) as a sampling function and ϕ(w) as the rejection function to effectively sample $E_{e}$. Specifically, for a given proton energy $E_{p}$, ν can be solely determined based on Equation (11), and hence, $F_{1}\left(\nu \right)$ and $F_{2}\left(\nu \right)$ are just numbers. We wrote them as $F_{1}$ and $F_{2}$ for simplicity in the following equations. Applying the direct inversion method, we could sample $w_{s}$ from f(w) as:

(23) $$w_{s}=\left(-F_{1}+2N_{c}\zeta +\sqrt{F_{1}^{2}+2F_{2}N_{c}\zeta -2F_{1}N_{c}\zeta }\right)/\left(F_{1}+F_{2}-2N_{c}\zeta \right).$$

Here, ζ∈[0,1) is a randomly sampled number and $N_{c}$ has the form of:

(24) $$N_{c}=\int_{0}^{w_{m}}f\left(w\right)dw=\left(w_{m}\left(F_{2}w_{m}+2F_{1}+F_{1}w_{m}\right)\right)/\left(2{\left(1+w_{m}\right)}^{2}\right).$$

We repeatedly sampled $w_{s}$ with Equation (23) and updated $\phi (w_{s})$ until we obtained $\phi \left(w_{s}\right)>\zeta^{\prime }$ with $\zeta^{\prime }$ a random number $\in [0,\;1/\left(1+e^{-\alpha w_{i}/v}\right)]$. Once reaching this stopping criterion, we accepted $w_{s}$ and computed $E_{e}$ as:

(25) $$E_{e}=w_{s}*B_{i_{0}}.$$

The polar scattering angle $\theta_{e}$ of the electron relative to the moving direction of the primary particle satisfied $\cos\theta_{e}=\sqrt{\frac{E_{e}}{4*T}}$ for $E_{e}>B_{i_{0}}$ and was uniformly distributed between zero and π otherwise [40]. The azimuth scattering angle was uniformly sampled between zero and 2π. The residual energy of the primary particle after ionization was ${E_{k}^{\prime }=E}_{k}-E_{e}-B_{i_{0}}$, and its polar scattering angle was zero.

If the sampled interaction $i_{0}$ belonged to the excitation category, there was no secondary electron emission, and we only needed to sample the energy loss *W* of the primary particle. In this case, the polar scattering angle for the primary particle was zero as well. When $E_{p}>500\;\mathrm{keV}$, we sampled *W* based on Equation (15). Noticing that $\frac{d\sigma_{i_{0}}^{exc}}{dW}\propto f_{i_{0}}\left(W\right)g\left(W\right)$, where $g\left(W\right)=\frac{1}{W}ln\left(\frac{4T}{W}\right)$, we then used $f_{i_{0}}\left(W\right)$ for the sampling of *W* and g(W) for rejection. For the ${\tilde{A}}^{1}B_{1}$ and ${\tilde{B}}^{1}A_{1}$ channels, $W_{s}$ can be directly sampled as:

(26) $$W_{s}=w_{i_{0}}+\frac{1}{\sqrt{2\alpha_{i_{0}}}}\zeta_{n},$$

where $\zeta_{n}$ is a random number following the standard normal distribution. As for plasma mode, we applied the direct inversion method to obtain $W_{s}$ as:

(27) $$W_{s}=w_{i_{0}}+\frac{1}{\alpha_{i_{0}}}\ln\left(\frac{u_{1}}{1-\alpha_{i_{0}}u_{1}N_{c}\zeta }-1\right).$$

Here, ζ is a random number ∈[0,1) and $N_{c}$ has the form of:

(28) $$N_{c}=\int_{W_{min}}^{W_{max}}f_{i_{0}}\left(W\right)dW=\frac{1}{\alpha_{i_{0}}}\left(\frac{1}{u_{1}}-\frac{1}{u_{2}}\right),$$

with $u_{1}=1+e^{\alpha_{i_{0}}\left(W_{min}-w_{i_{0}}\right)}$ and $u_{2}=1+e^{\alpha_{i_{0}}\left(W_{max}-w_{i_{0}}\right)}$. We repeated the sampling of $W_{s}$ and updating $g(W_{s})$ until we obtained $g\left(W_{s}\right)>\zeta^{\prime }$ with $\zeta^{\prime }$ a random number $\in [0,\frac{1}{W_{min}}\ln\left(\frac{4T}{W_{min}}\right)]$. The residual energy of the primary particle after excitation was then $E_{k}^{\prime }=E_{k}-W_{s}$. When $E_{p}\leq 500\;\mathrm{keV}$, the energy loss $E_{i_{0}}$ was directly obtainable from the discrete energy spectrum [37]. The residual energy of the primary particle was then ${E_{k}^{\prime }=E}_{k}-E_{i_{0}}$.

After transporting the primary particle with one step and simulating its interaction with one water molecule, we updated $E_{k}$ with $E_{k}^{\prime }$ and started the next round of transport sampling until the kinetic energy of the primary particle reached the cutoff energy or ran out in the simulation region. During the process, all secondary electrons were stored in a global stack to be further simulated using our previously developed kernel in charge of electron transport, the physics models that covered the electron spectrum as low as a few eV [9]. The ionized and excited water molecules were also tagged for further analysis.

#### 2.3.2. Concurrent Transport

In the concurrent transport picture, we simulated the reactions among radicals and DNA and the diffusion of the radicals in a step-by-step manner. Considering the complex structure of DNA and the possibly different checking frequencies for radical–DNA interactions and radical–radical reactions depending on the value of $t_{i}$, we utilized two GPU kernels for the chemical stage transport in the presence of DNA. One GPU kernel was responsible for the interactions between radicals and DNA, and the other kernel was in charge of the radical–radical reactions and the diffusion of the radicals.

For the GPU kernel managing the reactions between radicals and DNA, each GPU thread was responsible for one radical. To obtain the possible reaction and reaction type between the radical and DNA, we needed to search the DNA geometry and compute the distances from the radical to the centers of the DNA basic structures (DNA base, sugar-phosphate group, and histone protein). The smallest distance $d_{min}$ was then compared to the reaction range of $R+R_{c}$ with *R* the radius of that DNA structure. $R_{c}=k/4\pi N_{A}D$ for reactions between the radical and DNA base or sugar-phosphate group, where *k* is the reaction rate, $N_{A}$ is the Avogadro constant, and *D* is the diffusion rate for the radical. For all considered radical species, $R_{c}$ was <1 nm. Due to the lack of experimental data for the reaction between radicals and the histone protein, we assumed that the radical was only absorbed when it hit the histone, and hence, we set $R_{c}=0$ for this case. If $d_{min}<R+R_{c}$, a reaction was recorded. Otherwise, the Brownian bridge method [9] was applied to compensate for possible reactions between the radical and DNA during the diffusion. As our DNA model was constructed with a huge amount of basic structures, it would be too time consuming to search the entire space to obtain the smallest distance from the radical to the DNA. To reduce the searching burden, we relied on the voxelized geometry of the DNA model and only performed the search at most on two voxels. Specifically, noticing that the outer boundary of the DNA chain was >2 nm away from all edges of the voxel it occupied [9] and $R_{c}<1$ nm for all reactions between the radical and DNA, this indicated that a radical could only react with those DNA structures in two special voxels: the current voxel in which it was located and the adjacent voxel having the surface closest to it. The latter voxel was not considered unless the radical was <2 nm from its closest surface. In this way, we reduced the searching burden significantly. Once a reaction was recorded, the radical was removed from the reactant list. If the reaction was with the DNA base or sugar-phosphate group, the reaction site was stored in a global stack for further analysis.

For the radical–radical reactions and radical diffusion, we continued to employ the GPU kernel developed in our previous work [9]. Each thread was in charge of one radical. To reduce the searching burden for mutual reactions, the entire space was divided into small grids with the grid size twice the largest reaction radius. This ensured that each radical only reacted with other radicals in the same or adjacent grids. The distances from the radical to other radicals were then computed and compared to the reaction radii to obtain whether a reaction would occur. If a reaction happened, the new products were placed, and radical–radical reactions were checked again. Otherwise, the radical was diffused by one step followed by the check for radical–radical reactions based on the Brownian bridge method.

At the beginning of the chemical stage, the GPU kernel for the DNA–radical reactions was executed to remove the radicals within the chromatin region from the subsequent chemical-stage simulation. This was followed by the launch of the GPU kernel in charge of the radical–radical reaction and radical diffusion. After that, we compared $t_{\mathrm{elap}}$, the time elapsed from the last execution of the former GPU kernel, to $t_{i}$. If $t_{\mathrm{elap}}\geq t_{i}$, the former and latter kernels were called in sequence. Otherwise, only the latter kernel was executed. The process was repeated until reaching the end of the chemical stage.

### 2.4. Simulation Setup

#### 2.4.1. Simulation Setup for the Transport of Protons and Heavy Ions

We performed a series of simulations to validate the physical-stage transport for protons and heavy ions. These included: (1) the computations of the cross-section, linear energy transfer (LET), and traveling range; (2) the validation of the energy spectrum of secondary electrons, the radial dose distribution, and the track structure; and (3) the evaluation of the DNA damage spectrum.

**Figure 1:**
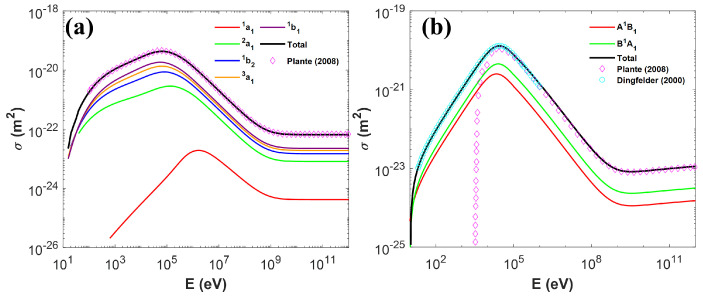
Total and partial cross-sections of (**a**) ionization and (**b**) excitation channels for protons with different energies.

We first calculated the total cross-section for both the ionization and excitation channels according to Equations (8) and (18), under the relativistic formats. The results were compared to Plante et al.’s [34] and Dingfelder et al.’s [35] works, as shown in Figure 1. Based on this, we calculated the track-length-averaged unrestricted LET for different ion species with their energy ranging from $0.1∼10^{4}\;\mathrm{MeV}\;{\mathrm{amu}}^{-1}$. For an ion with energy $E_{k}$, we sampled the energy loss of primary particles $\varepsilon_{i}$ and the free-fly distance $s_{i}$. We then repeated the simulation ${N=10}^{5}$ times and computed the length-averaged unrestricted LET as:

(29) $$LET=\sum_{i=1}^{N}\frac{\varepsilon_{i}}{s_{i}}\cdot \frac{s_{i}}{\sum_{j=1}^{N}s_{j}}=\frac{\sum_{i=1}^{N}\varepsilon_{i}}{\sum_{j=1}^{N}s_{j}}.$$

**Figure 2:**
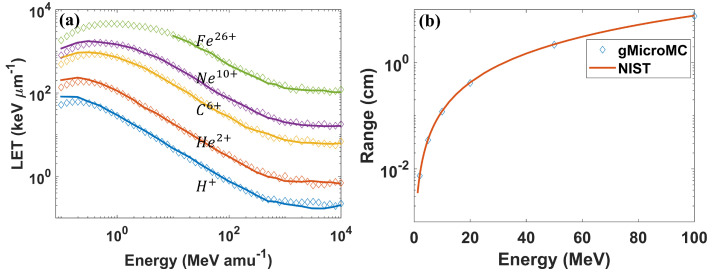
(**a**) The unrestricted LETs for different ions with different energies. The unit amu${}^{-1}$ means per nucleon. Solid lines represent data extracted from Plante et al.’s work, while data with diamond symbols are from our simulation with gMicroMC. (**b**) The simulated proton range for different energies.

We compared the LETs to those reported by Plante et al. [34]. After that, we simulated the proton range by tracking its starting and ending positions for a proton energy of 0.1∼100MeV and compared this to the NIST data. We show both results in Figure 2.

**Figure 3:**
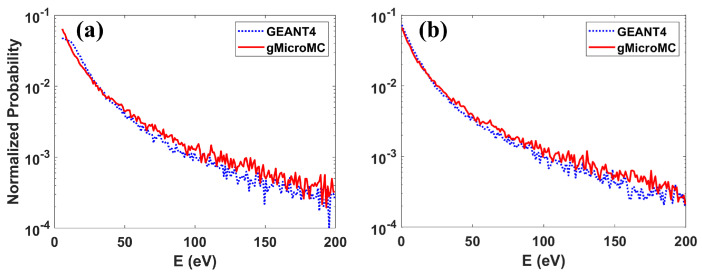
Secondary electron spectrum for (**a**) a 5 MeV proton and (**b**) a 4 MeV alpha particle.

As for the validation of the energy spectrum of secondary electrons, we simulated the interactions of a 5 MeV proton and 4 MeV alpha particles with a liquid water target, recorded the energy of the secondary electrons, and compared it to those obtained with the GEANT4-DNA simulation [43] (GEANT4 Version 10.5.1). The result is plotted in Figure 3. As for the radial dose distribution, we transported 5 and 10 MeV protons within a water slab of 10 µm in thickness and infinitely long at the other two dimensions and analyzed the dose distribution within a thin slice 4 to 6 µm away from the proton starting point along the thickness direction. We accounted for the dose distributed in an annular ring with inner and outer radii of *r* and r+Δr as the dose at radius *r*. We set Δr = 1 nm, the same as that used in Wang et al.’s work [44]. We repeated the simulation $10^{5}$ times, averaged the obtained radial dose, and compared it with that reported in Wang et al.’s work [44]. Finally, we show a representative physical track structure for a 5 MeV proton in Figure 5, including the track for both the primary proton and secondary electrons.

We used the lymphocyte nucleus model developed in our previous work for this evaluation study [9]. We initiated a proton emission plane of 11×11 µm^2^ and 5.5 µm away from the center of the cell nucleus for two proton energies, 0.5 and 0.9 MeV. For each energy, we randomly sampled the proton position at the emission plane and its momentum towards the positive z direction, transported the proton until reaching a cutoff energy of 1 keV, and recorded the dose inside the cell nucleus. We repeated the simulation until reaching the accumulated dose of 2 Gy. After that, we simulated the physio-chemical and chemical stage with a chemical stage duration of $t_{c}$ = 1 ns. We then applied the overlay method to obtain the DNA damage sites and grouped them into DNA Single-Strand Breaks (SSBs) and Double-Strand Breaks (DSBs) [29]. The result was compared to Nikjoo et al.’s work with the KURBUC model [45] and shown in Table 4.

#### 2.4.2. Simulation Setup for Concurrent Transport

We studied the impact of $t_{i}$ on the radical yields. We simulated the cases with a chemical stage duration of $t_{c}=1$, 10 ns, and 1 µs and $t_{i}$ from 1 ps to 1 µs with an increment of one at the logarithmic ten scale. Again, the lymphocyte cell nucleus with a radius of 5.5 µm was used as the Region Of Interest (ROI). As for the radical yield, we transported a 4.5 keV electron with its initial position randomly sampled inside the ROI and its direction towards the ROI center. We then took the generated radicals as inputs for the chemical-stage simulation. The final G values for the $e_{h}$, OH·, H·, and $H_{2}O_{2}$ radicals were recorded. We repeated the simulation 100 times to reduce the statistical uncertainties and reported the averaged G values over all the simulations. The results are shown in Figure 6.

We also computed the DNA damage as a function of the incident proton energy and the chemical stage duration under the concurrent DNA transport frame. A proton energy $E_{k}$ of 0.5, 0.6, 0.8, 1.0, 1.5, 2, 5 10, 20, and 50 MeV and a chemical stage duration $t_{c}$ of 1, 2.5, and 10 ns were considered, following the parameters used in Zhu et al.’s work [42]. We initiated the proton on a spherical shell with a radius of 5.5 µm and shot it randomly towards the inner space of the sphere. We repeated the simulation until having the accumulated dose in the sphere of 1 Gy. We then simulated the chemical stage with DNA concurrent transport ($t_{i}=1$ ps) and computed the total DSB yield. For each proton energy, using the DSB yield at $t_{c}=1\;ns$ as a reference, we defined $R\left(t\right)=N_{DSB}\left(t_{c}=t\right)/N_{DSB}\left(t_{c}=1\;ns\right)$ to represent the relative DSB yields at $t_{c}=t$. For each pair of $E_{k}$ and $t_{c}$, we ran the simulation 20 times and computed the mean and standard deviation for the relative DSB yield. We then compared the data with $t_{c}=2.5\;\mathrm{ns}$ (R(2.5)) and 10 ns (R(10)) to Zhu et al.’s work [42] and show the results in Figure 7.

## 3. Results

### 3.1. Validation of Development for Protons and Heavy Ions

Figure 1 presents the total and partial cross-sections for ionization and excitation as a function of incident proton energy. From Figure 1a, the total cross-section for ionization from our simulation agreed well with that from Plante et al.’s work [34]. From Figure 1b, for a proton energy >500keV, our simulated total cross-section for excitation matched that from Plante et al.’s work [34]. As for the slow proton, it followed that from Dingfelder et al.’s work [35]. The results revealed that the ionization model and the two-stage excitation model were successfully implemented as expected.

In Figure 1b, we noticed a dramatic drop-off of the total excitation cross-section at around 10 keV for the Plante model. This is due to the cross-section formula shown in Equation (15) depending on the scaled energy $T=\frac{m}{M}E_{k}$. When $E_{k}$ drops below 10 keV, *T* is too small to excite even the lowest excitation channel ($j={\tilde{A}}^{1}B_{1}$). After replacing it with Dingfelder’s model (Equation (19)) at the low-energy region, the excitation cross-section drops much more slowly. Considering that the low-energy proton largely appears after the Bragg peak, a proper excitation model could be important for the distal dose computation in proton therapy.

The calculated unrestricted LETs for different ions are plotted in Figure 2a. They agreed well with Plante et al.’s work for ions with an energy greater than 0.5 MeV per nucleon. At the low energy range, LETs from our simulation were lower than those from Plante et al.’s work, which can be explained by the different excitation models used in the two simulations. As shown in Figure 1b, the excitation cross-section from our work was higher than that from Plante et al.’s work at the low energy range, resulting in a higher sampling rate of excitation interactions in our simulation. Considering that the energy loss from an excitation event was typically smaller than that from an ionization event (Table 1), a higher sampling of excitation could result in a lower LET. In Figure 2b, we show the proton range from our simulation and its comparison with the NIST data. As is shown, our simulation result matched well with the NIST PSTAR data (https://physics.nist.gov/PhysRefData/Star/Text/PSTAR.html (accessed on 17 June 2021)), with the relative difference smaller than 1%.

Figure 3 shows the energy spectrum of secondary electrons generated from a 5 MeV proton and a 4 MeV alpha particle. From the figure, the yielding rates of secondary electrons dropped quickly along with the increase of the electron energy. For the entire plot, our simulated results agreed well with that from GEANT4-DNA. We did not compare the spectrum for electron energy greater than 200 eV due to a too low yielding rate and the consequent large uncertainty.

**Figure 4:**
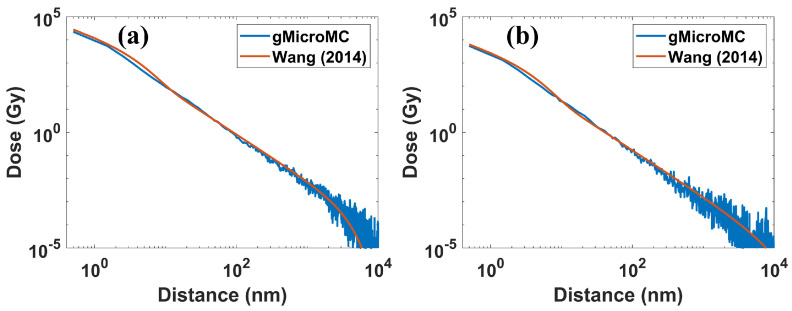
Radial dose distributions for (**a**) 10 MeV and (**b**) 50 MeV protons.

In Figure 4, we see the radial dose distributions for 10 and 50 MeV protons from ours and Wang et al.’s work (Equations (1)–(7) in [44]) under the same setup. As is shown, the two curves matched quite well, although the curve from our simulation suffered a relatively large statistical fluctuation for the regions >1000 nm from the primary track axis. Figure 3 and Figure 4 together validated the energy spectrum and angular distribution of secondary electrons from our simulation, furthering proving the successful implementation of the transport models for protons and heavy ions.

**Figure 5:**
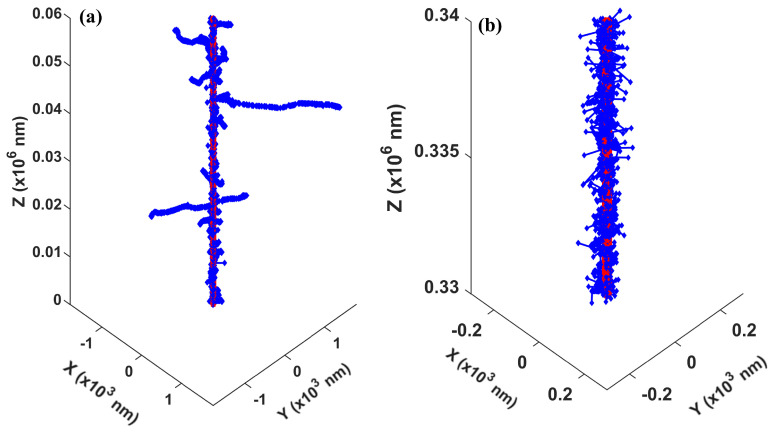
A representative track structure for a 5 MeV proton at the entrance part (**a**) and in the Bragg peak region (**b**). The proton was emitted along the positive *Z* direction. Red and blue dots represent the energy depositions by the proton and secondary electrons, respectively. Note: in the two subplots, we kept the same aspect ratio between the z and x/y axes, but plotted them with different ranges.

We then present a track structure for a 5 MeV primary proton and its produced secondary electrons in liquid water in Figure 5. For simplicity, we only present the entrance (Figure 5a) and Bragg peak (Figure 5b) regions. At the entrance region, the secondary electron tracks were quite sparse. In contrast, they were much denser in the Bragg peak region. In addition, the electron track lengths were shorter in the Bragg peak region. This was mainly due to the kinetic energy of the proton being much higher at the entrance than the Bragg peak region. This led to a smaller total cross-section and a longer interval between the production of secondary electrons. Plus, high-energy electrons (Equation (23)) would be favored when the proton energy is high. In general, most of the electrons travel a tiny distance before being locally deposited, forming the dense blue area around the central proton line, and hence a high radial dose distribution in the regions with small radii (Figure 4).

Finally, in Table 4, we report the DNA damages in the form of DSBs induced by 0.5 and 0.9 MeV protons. The results from our simulation were compared with those from Nikjoo et al.’s work with the KURBUC model [45]. The difference was found within 10%.

### 3.2. Validation of Concurrent Transport

**Figure 6:**
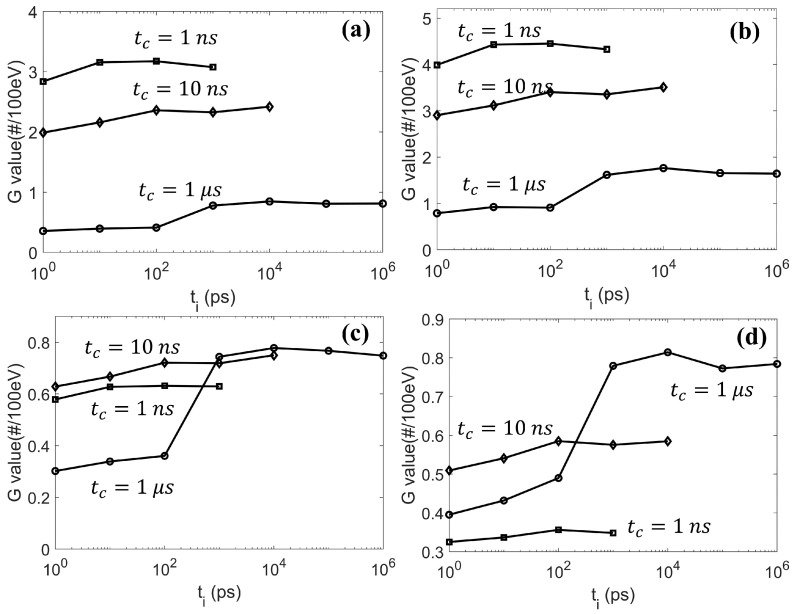
The yields of (**a**) $e_{h}$, (**b**) OH·, (**c**) H·, and (**d**) $H_{2}O_{2}$ chemical species at different checking time intervals $t_{i}$ and chemical stage durations $t_{c}$.

As for the validation of the concurrent DNA transport module, we first studied the influence of different checking time intervals $t_{i}$ and chemical stage durations $t_{c}$ on the yields of different radicals. As shown in Figure 6, at a fixed $t_{i}$, the yields of the $e_{h}$ and OH· radicals reduced when $t_{c}$ increased. This was because longer $t_{c}$ enabled more reactions among radicals and DNA. For the $e_{h}$ and OH· radicals, these reactions were mainly consumption channels, resulting in a reduced yield rate when $t_{c}$ increased. In contrast, although the presence of DNA also consumed H· and $H_{2}O_{2}$ radicals, reactions among radicals could contribute positively to the yields of these two radicals. Hence, the production of the H· and $H_{2}O_{2}$ radicals could be dependent on $t_{c}$ in a more complex way. In addition, at a fixed $t_{c}$, varying $t_{i}$ from 1 ps to $t_{c}$ transformed the simulation from the concurrent method to the overlay method. All lines were connected smoothly, and the G value with $t_{i}=t_{c}$ matched with that in our previous publication under the overlay method, indicating the self-consistency of the concurrent DNA transport in gMicroMC.

**Figure 7:**
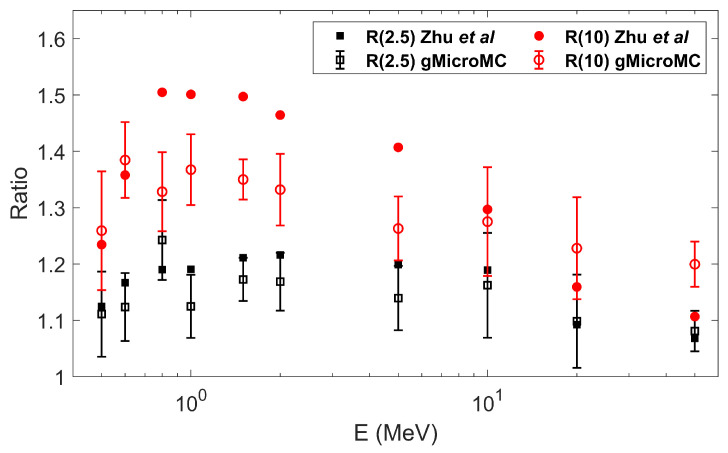
The relative DSB yields at different chemical stage durations $t_{c}$ and different proton energies with $R\left(t\right)=\frac{DSB\left(t_{c}=t\right)}{DSB\left(t_{c}=1\;ns\right)}$. The data from gMicroMC simulation were compared to that from Zhu et al.’s work [42].

After examining the self-consistency of the developed concurrent DNA transport method, we comprehensively studied the DSB yields as a function of proton energy $E_{k}$ and chemical stage duration $t_{c}$. The results were compared to Zhu et al.’s work [42] and are shown in Figure 7. From the figure, all data points had relative DSB yields >1, and the R(10) values were larger than R(2.5) for the same $E_{k}$. This indicated the DSB yields increased when the chemical stage expanded from 1 ns to 10 ns under the concurrent transport frame. The reason was that the longer the chemical stage lasted, the more checks between radicals and DNA were performed, and hence, more DSBs could be formed. Along with the increase of the proton energy, the relative DSB yields exhibited a maximum in the middle energy range. Comparing the data from our simulation to that from Zhu et al.’s work, the trends generally agreed, especially for the R(2.5) data. Some larger discrepancies existed for the R(10) values, which could be explained partially by the different radical diffusion rates and different DNA geometries applied in the two works. For example, the diffusion rate of the OH· radical was larger in our package. This could make OH· diffuse longer and experience a higher scavenging rate from the histone protein within one diffusion step. In addition, a larger diffusion rate could result in a smaller reaction radius between OH· and the DNA sugar-phosphate moiety. Both led to a reduced DSB yield. The longer the $t_{c}$ was, the more reduction effect it could create, such that we would obtain a smaller relative DSB yield than that from Zhu et al.’s work for the R(10) data than for the R(2.5) data.

### 3.3. Computational Efficiency

After evaluating the two newly developed features of gMicroMC by comparing to the NIST data or simulation results from other packages, it was important to evaluate the time performance of the new modules for practical applications. In Table 5, we show the simulation time for the physical transport of 1, 10, and 100 protons at 1 and 10 MeV with gMicroMC on a single GPU card (Nvidia V100). As can be seen, it only took 2 and 4 s to transport a single proton with an initial kinetic energy of 1 and 10 MeV. In contrast, it could take multiple hundreds to thousands of seconds to perform similar simulations with single-CPU-based packages, showing the high efficiency of gMicroMC. It is also important to point out that when raising the proton numbers from one to one-hundred, the simulation time only increased by <5 and 10 folds for 1 and 10 MeV protons, respectively. This feature was against the linearly increasing behavior for typical CPU-based simulations, making gMicroMC especially suitable for large-scale simulations. Actually, when the proton number was small, the parallel computing capacity of the GPU was far from being exhausted when launching the kernel for the primary particle transport, such that increasing the proton number, the running time would not significantly increase.

As for the simulation time of the concurrent transport ($t_{i}=1$ ps) in the chemical stage, it could be affected by many parameters, such as the number of radicals, the chemical stage duration, and the DNA complexity. Here, we reported the simulation time for a representative case. Considering that the number of radicals produced from one a 100 keV electron or a 1 MeV proton was roughly $10^{5}$ and the longest chemical stage duration used in the overlay method was ∼1 µs, we set the initial number of radicals ${N_{i}=10}^{5}$ and chemical stage duration $t_{c}=$ 1 µs in our simulation. We also included our DNA model containing of ∼$6.2\times 10^{9}$ bps for a human lymphocyte cell nucleus in the simulation, the complexity of which is high. The simulation time was found to be 470 s with gMicroMC on a single GPU. Compared to the simulation time of 31 s under the overlay scheme for gMicroMC, the concurrent transport frame was still quite efficient since the simulation became much more sophisticated with the presence of DNA. This indicated that gMicroMC can be applied in simulations with realistic settings. In comparison, restrictions on the reaction region and time duration, etc., are typically required in other packages for memory- or time-saving purpose [22,23].

## 4. Discussion

This is a continuous development work on gMicroMC. In this work, we successfully implemented the physical transport for proton and heavy ions and the concurrent transport of radicals and DNA in the chemical stage. For the former implementation, we considered the ionization, excitation, and charge effect during the transport and performed a series of case studies to validate the development. The obtained results matched well with the literature reports. We then computed the DNA DSBs for two proton energies, and the results agreed with those computed with the KURBUC model in Nikjoo et al.’s work [45] within 10%. As for the latter, we considered the interaction of radicals with the DNA, histone protein, base, and sugar-phosphate groups during the chemical transport. To validate it, we first performed a self-consistency check for the evolution of chemical radicals and induced DNA DSBs under different checking frequencies for radical–DNA interactions. The result showed the high self-consistency of the developed module. We then performed a comprehensive study of the DSB yields as a function of the chemical stage duration and incident particle energies under the DNA concurrent transport frame. The results generally followed that from Zhu et al.’s work. The use of the GPU made the code have high computational efficiency. Running gMicroMC on a single GPU card, it took only 41 s to transport 100 protons with a kinetic energy of 10 MeV and around 8 min to transport $10^{5}$ radicals up to 1 µs with the presence of a DNA model containing ∼$6.2\times 10^{9}$ base pairs. The high computational performance of gMicroMC makes it suitable to simulate complex radiation scenarios such as proton FLASH radiotherapy, which is our next work. To benefit the community, we are also working on releasing the newly developed features of gMicroMC on GitHub (https://github.com/utaresearch/gMicroMC (accessed on 17 June 2021)).

Despite the above success, there are also some limitations to our current development. In the physical transport of protons and heavy ions, we ignored the nuclear inelastic interaction and the nuclear and electromagnetic elastic scattering. Among them, the nuclear inelastic interaction can fragment the target and/or projectile nuclei, which is a main factor to remove primaries from the projectile beam. However, due to the complexity of the fragmentation process and its products, this process is typically not directly included in any mechanistic MC simulation tools at this moment [46]. Since this work mainly focused on the novel GPU implementation of existing models, it will be our next work for a possible inclusion of the nuclear inelastic scattering process. As for the elastic scattering, it could change the direction of the primaries, hence affecting the track structure and radical dose distributions. However, elastic scattering mainly dominants interactions between the proton and water medium at a very low energy range, and the scattering angle is typically small [46,47]. Hence, we expect it will not affect the accuracy of the DNA damage computation greatly. Considering the powerful computational capacity of the GPU, it is promising to consider a more complete modeling of the physical interactions between ions and water molecules, making gMicroMC versatile for advanced applications.

It is also worth pointing out that we applied a low-energy five-pathway model and a high-energy three-pathway model to simulate the proton-induced excitation of a water molecule such that both very-low-energy and relativistic situations could be covered. Yet, this could cause a concern that some excitation pathways could be discontinuous at the model switching point. A previous study showed that the low-energy model could be extended up to 80 MeV with some proper parameter fitting [34]. We hence made it an option in our package to only apply Equation (19) to model the excitation process up to 80 MeV. In addition, for the two-model picture, although we currently set the model switching point at 500 keV to distinguish the slow and fast proton behavior following the same logic as stated in [35], it deserves further study to investigate its impact on the subsequent radical yielding process.

Another important procedure that could affect the computational accuracy of the proton- and heavy ion-induced DNA damage is the modeling of the secondary electron transport, especially for the low-energy electrons (sub-keV range). Previous studies revealed their critical importance in determining the initial distribution of radicals and the consequent DNA damage patterns [3]. However, due to the lack of sufficient experimental data, uncertainties about the simulation results could be introduced by the inaccurate modeling of this process. For instance, with different models adopted in gMicroMC and Geant4-DNA, the maximal discrepancies of the track length and penetration depth for electrons below 1 keV computed by the two packages were around 10 and 4 nm, respectively [9]. In recent years, there has been much effort contributing to improving the accuracy of describing the low-energy electron transport process [11,12,13,14,15,48]. However, more efforts are required to fully elucidate this problem.

In addition, as discussed in our previous study [29], the cross-section used to simulate the ionization and excitation processes from electrons could significantly impact the accuracy of the finally obtained DSB yields. In the case of protons and heavy ions, due to the lack of experimental data, the cross-sections and models could also be associated with large uncertainties, causing concerns about the robustness of the simulation results. To reduce these uncertainties, there have been multiple experiments and models [13,49,50,51,52,53,54,55,56] developed in a more elaborate fashion. Yet, more studies are needed to more thoroughly understand the relevant processes.

**Figure 8:**
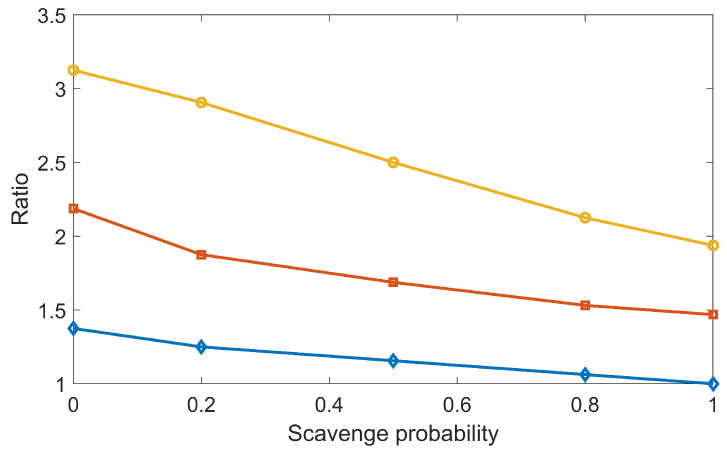
Ratio of DSB yields with different scavenge probabilities.

In our previous study [29] with the overlay method, the DSB yields reduced when the chemical stage duration enlarged, which was against the trend obtained in this work with the concurrent DNA transport method (Figure 7). This was due to the fact that in the overlay method, the radical–DNA interaction was only simulated after the chemical-stage simulation. The longer the chemical stage lasted, the more the OH· radicals were consumed during the mutual radical reactions, and the fewer the DSBs could be formed. Nonetheless, the controversial behavior between the concurrent and overlay frames reminds us to carefully check the parameters used in our simulation. One such parameter is the scavenging probability from histone proteins to chemical radicals, the value of which has not been well regulated by current experiments. We performed a new simulation to study its impact on DSB yield by gradually reducing the scavenging probability $P_{s}$ from one to zero. Here, $P_{s}=1$ means the total scavenging probability once radicals hit the histone proteins. Taking the DSB yields at $P_{s}=1$ and $t_{c}=1\;ns$ as a reference, we computed the relative DSB yields at other $P_{s}$s and $t_{c}$s. The results are shown in Figure 8. Clearly, DSB yields increased when $P_{s}$ decreased. However, even under the same $P_{s}$, when $t_{c}$ differed, $P_{s}$ could have different impacts on the relative DSB yield. For instance, the relative DSB yield was 1.4 for $t_{c}=1\;ns$, while it was 3.1 for $t_{c}$ = 1 µs when $P_{s}=0$. This indicated that to make the simulation tool robust for various applications, we need to apply a proper cutoff to $t_{c}$ and a detailed coordination of multiple parameters used in the chemical-stage simulation. This should be considered in future studies.

In our development of the concurrent transport module, we used a complete set of cellular DNA at the base-pair resolution to simulate the radical–DNA interactions. Previous studies have incorporated other DNA models, including the simple cylinder DNA [3], other cellular DNAs [7,42,57], and the DNA model at the atomic resolution [58,59]. Due to the different simulation setups and different DNA structures adopted, the absolute DSB yields from different studies were typically non-comparable [60]. However, there were some common trends shared among different studies. For example, the DSB yields were found increasing and then decreasing when the LETs increased. The maximal yields of DSB occurred at the LET value around 60 keV/µm in [57] and around 27.2 keV/µm (the LET value for 1 MeV protons [22]) for gMicroMC and TOPAS-nBio, respectively. These consistencies motivated further studies with the concurrent simulation scheme while the expense was the lowered simulation efficiency. Our adoption of the GPU-based acceleration could shed light on this issue based on experiences from previous studies [9,27,28,61].

**Figure 9:**
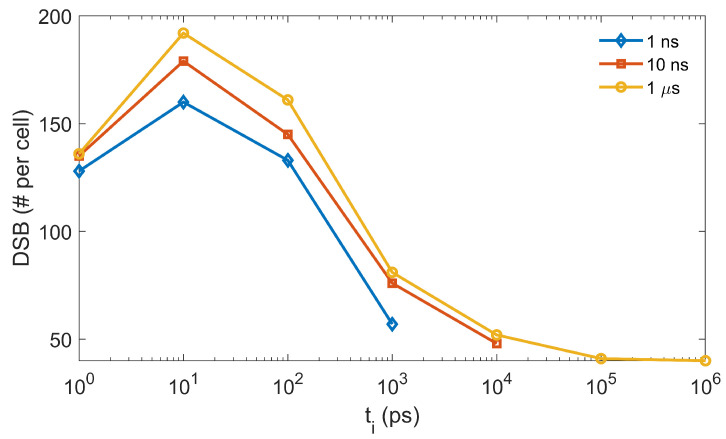
The yields of DSB at different $t_{i}$ and $t_{c}$ from the gMicroMC simulation.

Finally, we performed a further study on the effect of $t_{i}$, which was introduced to balance the simulation efficiency and accuracy. In the *Results* Section, we showed the obvious impact of $t_{i}$ on the radical evolution. It would be interesting to see how it could consequently affect the final DNA damage. As the DSB is widely accepted as the most important factor in cell death, it is thus reasonable to use the DSB as a metric to evaluate the impact of $t_{i}$. We initiated a 4.5 keV electron with its position randomly sampled inside a sphere of radius 6.1 µm and its direction following the isotropic distribution. The sphere was concentric with the cell nucleus of our DNA model. We repeated the simulation until the accumulated dose inside the cell nucleus region reached 1 Gy, equivalent to simulating ∼ 2000 electrons. The generated radicals were then transported in the chemical stage along with considering the radical–DNA reactions. We calculated the resulting DNA DSBs as a function of $t_{i}$ and show these in Figure 9. For the three $t_{c}$ studied, the DSB lines showed a similar trend when $t_{i}$ increased. The maximal DSB was obtained at $t_{i}=10$ ps. The result could be interpreted as follows. At the beginning of the chemical stage, all generated radicals had a relatively dense distribution. When $t_{i}$ slightly increased, the OH· radical could diffuse a longer distance away from its initial position before it reacted with the DNA, while its reaction probability with other radicals was not greatly affected. Hence, a sparser, but equivalent (or slightly reduced) number of DNA damage sites could be formed, which could lead to more generations of simple DSBs (composed of two damage sites) rather than the DSB+ (composed of multiple damage sites). In this way, the total DSB yields could increase. However, along with the further increase of $t_{i}$, the checking frequency for radical–radical reactions would be much higher than that for radical–DNA reactions. This could lead to a higher consumption of OH· radicals through radical–radical reactions than through DNA damaging reactions, which resulted in a reduced DSB yield.

## 5. Conclusions

We successfully developed and validated two new features in gMicroMC, the transport of protons and heavy ions in the physical stage and the concurrent transport of DNA in the chemical stage. We implemented the two features on a GPU parallel computing platform, resulting in a remarkable time performance. The physical transport of 100 protons with an initial kinetic energy of 10 MeV could be finished in s. The chemical simulation with concurrent DNA transport was far more complex, but it still only took a few minutes to run a representative case. The two newly developed features in gMicroMC that had both high accuracy and efficiency gave gMicroMC the high promise of solving large-scale problems in active radiation research areas.

## Figures and Tables

**Table 1:** Parameters used in this work for Equations (1)–(7). Data were extracted from [35,38].

Parameter	Inner Orbitals		External Orbitals		
${}^{1}a_{1}$	${}^{2}a_{1}$	${}^{1}b_{2}$	${}^{3}a_{1}$	${}^{1}b_{1}$
$A_{1}$	1.25	1.25	1.02	1.02	1.02
$B_{1}$	0.5	0.5	82	82	82
$C_{1}$	1	1	0.45	0.45	0.45
$D_{1}$	1	1	−0.80	−0.80	−0.80
$E_{1}$	3	3	0.38	0.38	0.38
$A_{2}$	1.1	1.1	1.07	1.07	1.07
$B_{2}$	1.3	1.3	14.6	14.6	14.6
$C_{2}$	1	1	0.6	0.6	0.6
$D_{2}$	0	0	0.04	0.04	0.04
α	0.66	0.66	0.64	0.64	0.64
$N_{i}$	2	2	2	2	2
$B_{i}$	539.7	32.2	18.55	14.73	12.61

**Table 2:** Parameters used in Equations (17).

*j*	${\tilde{A}}^{1}B_{1}$	${\tilde{B}}^{1}A_{1}$	Plasma Mode
$f_{j}^{0}$	0.0187	0.0157	0.7843
$\alpha_{j}$	3 (eV${}^{-2}$)	1 (eV${}^{-2}$)	0.6 (eV${}^{-1}$)
$w_{j}$	8.4	10.1	21.3

**Table 3:** Reaction rates (${\times 10}^{9}\;L\cdot mol^{-1}\cdot s^{-1}$) used in gMicroMC for concurrent DNA transport [41].

Radicals	A	G	C	T	DNA Base	DNA Sugar-Phosphate Group
OH·	6.1	9.2	6.4	6.1	6.95	1.9
$e_{h}$	9	14	18	13	13.5	−1 *

^*^ A negative value means no reaction between the radical and the DNA substructure.

**Table 4:** The DSB yields (number per Gy per Gbp) obtained under the overlay method for two proton energies.

Energy (MeV)	from gMicroMC	from Nikjoo’s Work
0.9	20.1	18.2
0.5	25.1	23.9

**Table 5:** The simulation time (s) of physically transporting 1, 10, and 100 protons for proton energies at 1 and 10 MeV by gMicroMC on a single GPU card.

Energy (MeV)	Number of Primary Protons		
	1	10	100
1	1.9	3.1	9.3
10	3.9	9.8	40.5

## Data Availability

Not applicable.

## Abbreviations

The following abbreviations are used in this manuscript:

GPU	Graphical Processing Unit
LET	Linear Energy Transfer
ROI	Region Of Interest
SDCS	Singly Differential Cross-Section
OER	Oxygen Enhancement Ratio
PSF	Phase Space File
